# Target Volume Delineation Using Diffusion-weighted Imaging for MR-guided Radiotherapy: A Case Series of Laryngeal Cancer Validated by Pathology

**DOI:** 10.7759/cureus.2465

**Published:** 2018-04-11

**Authors:** Hans Ligtenberg, Tim Schakel, Jan Willem Dankbaar, Lilian N Ruiter, Boris Peltenburg, Stefan M Willems, Nicolien Kasperts, Chris H J Terhaard, Cornelis P J Raaijmakers, Marielle E P Philippens

**Affiliations:** 1 Department of Radiotherapy, University Medical Center Utrecht, Utrecht, NLD; 2 Department of Radiology, University Medical Center Utrecht, Utrecht, NLD; 3 Department of Pathology, University Medical Center Utrecht, Utrecht, NLD

**Keywords:** dwi, radiotherapy, larynx, validation, pathology, gtv, target delineation

## Abstract

In radiotherapy treatment planning, tumor delineation based on diffusion-weighted imaging (DWI) by magnetic resonance imaging (MRI) is a promising technique. MR-only-based target definition becomes important with the recent development of MRI integrated radiotherapy treatment modalities. In this case series, DWI-based gross tumor volume (GTV) was validated using pathology and compared with a clinical GTV based on computed tomography (CT) imaging and MRI.

This case series includes three patients with a laryngeal tumor. Prior to total laryngectomy (TLE), imaging was performed on CT and MRI, including a DWI scan. After TLE, the surgical specimen was processed and cut into 3-mm thick slices. The tumor was delineated on hematoxylin-eosin (HE) stained sections by a pathologist (tumor_HE_). This pathological imaging, including the tumor_HE_ delineation, was three-dimensionally reconstructed and registered to the imaging. The GTV was delineated by a radiation oncologist based on CT and MR imaging (GTV_clinical_) and semi-automatically delineated based on DWI (GTV_DWI_).

The microscopic tumor extent outside the GTV_DWI_ contour was 3.0 mm, 2.7 mm, and 11.3 mm for cases I, II, and III, respectively. The microscopic tumor extent outside the GTV_clinical_ was 7.5 mm, 2.1 mm, and 1.5 mm for cases I, II, and III, respectively. The tumor, on histology, was covered by the GTVs for 80%, 74%, and 31% (GTV_DWI_) and 73%, 72%, and 89% (GTV_clinical_) for the three subsequent cases, respectively. The GTV_DWI_ resembled the tumor_HE_ more than the GTV_clinical_ in case I and case II. In case III, GTV_DWI_ missed the caudal part of the tumor that was included in the clinical delineation due to a lack of contrast and the heterogeneous signal intensity of the tumor in DWI.

In this case series, we showed the potential of DWI for MR-guided radiotherapy treatment if a clear contrast is visible. DWI-based GTV delineation might be a fast alternative to manual delineation, which could speed up the on-table target definition using an MRI-linac system. A larger case series is needed to verify these results.

## Introduction

The concept of improved radiation treatment with a magnetic resonance imaging (MRI)-linac is based on a better targeting of the tumor and a sparing of organs at risk (OARs) by better visualization using MRI [1]. MRI-only based target definition will become very important with the need for online target definition. Therefore, precise and accurate target definition using MRI is crucial for successful and high-quality radiation treatment. Incorrect target definition can result in an undertreatment of tumor tissue, which might result in reduced local control, whereas an overestimation of the target can lead to increased normal tissue complications [2-3]. This is especially the case for head-and-neck cancer, where the primary tumor, typically, is located in close proximity to the organs at risk.

Conventionally, computed tomography (CT) is used for gross tumor volume (GTV) delineation, whereas positron emission tomography (PET) and magnetic resonance imaging (MRI) are used to provide complementary information. However, the correct interpretation of the various images remains challenging, despite the amount of imaging data available. This is demonstrated by variations in GTV delineations based on CT, MRI, and PET, as well as high interobserver variation [2,4]. GTV delineation validation studies using surgical specimens and (histo)pathology can evaluate the accuracy of these delineations and provide insight into the correct interpretation of the images [5-6]. Validation is especially important for functional imaging because of the lack of anatomical information and the limited experience.

Recently, diffusion-weighted MRI (DWI) has gained interest for GTV delineation [7]. DWI provides information on tissue microanatomy by a reflection of the local mobility of water molecules. Consequently, tumors with their high cell density might be distinguished from healthy tissue, resulting in high image contrast. The conventional DWI acquisition method is prone to geometric distortions, especially near air cavities. However, using a different acquisition scheme, geometrically accurate diffusion-weighted images can be acquired in a radiotherapy simulation setup [8]. This technique might pave the way for fast semi-automatic tumor segmentation based on MRI. As an MRI-only-based target definition will become more prevalent with the recent development of MRI-linear accelerators (MRI-linac) [1], the need for online target definition will increase.

As this geometrically accurate DWI technique has a potential for (semi-)automatic tumor segmentation, we validated tumor target volumes defined on DWI with histopathology in laryngeal tumors in this case series. For a comparison with current clinical practice, target volume delineations based on a DWI were compared with standard clinical target volume delineations.

## Case presentation

Technical details

In this study, three cases with a primary T4a laryngeal squamous cell carcinoma are presented, which underwent a TLE. Prior to surgery, CT imaging was performed. Various MR scans were acquired at 3.0 T (Ingenia, Philips Healthcare, The Netherlands), including a T1-weighted scan with gadolinium contrast, a T2-weighted scan, and a geometrically accurate turbo-spin-echo (TSE)-DWI sequence [8] with three b-values (s/mm^2^): b0, b200, and b800. A corresponding apparent diffusion coefficient (ADC) map was calculated. All imaging was performed in the radiotherapy treatment position using a five-point head-and-shoulder mask.

First, a clinical GTV delineation was performed based on the CT and MRI scans. Second, a semi-automatic tumor segmentation was performed based on a 50% signal-intensity threshold in the b800 DWI scan. Subsequently, regions of high ADC, typically at the edge of the tumors, were excluded manually from the GTV.

Following TLE, the surgical specimen was processed and reconstructed, as described by Ligtenberg et al. [6] and Caldas-Magalhaes et al. [9] and summarized in the introduction. On the obtained HE sections, a head-and-neck pathologist digitally delineated the tumor tissue (tumor_HE_). A three-dimensional specimen was reconstructed from the HE sections. All imaging, including CT, MRI, and the 3D-reconstructed specimen, was rigidly registered with each other while taking into account shrinkage due to the fixation and dehydration of the surgical specimen.

The clinical GTV delineation and DWI-based GTV delineation were compared with each other and with a histopathology-based tumor_HE_ delineation. The percentage overlap of the GTVs with the tumor_HE_ and the extent of the tumor outside the delineated GTVs was determined. Furthermore, the conformity index of the GTV with tumor_HE_ was determined, which is the intersection volume of the GTV with the tumor_HE_, divided by the encompassing volume of the GTV together with the tumor_HE_. To determine the extent of the microscopic tumor growth outside the GTV, the 95th percentile Hausdorff distance between tumor_HE_ and the intersection of tumor_HE_ with the GTV was determined.

Case I

The first case involves a male, age 66 years, with a cT4aN0M0 laryngeal carcinoma, which was pathologically confirmed. The interval between surgery and pre-treatment CT and MRI was nine and two days, respectively.

On the imaging, an enhanced mass with restricted diffusion arising from the right true vocal cord was visible. The tumor showed a clear contrast on the b800 DWI scan with the surrounding tissue with homogeneous and high-diffusion restriction and corresponding low ADC values on the ADC map. Also, on the T1-weighted MRI, the gadolinium contrast uptake was homogeneous, with locally increased uptake adjacent to the tumor. The T2-weighted image showed contrast with the surrounding tissue, but a heterogeneous signal was observed within the tumor.

The maximum diameter was 3.5 cm extending cranially into the false vocal cord and caudally into the subglottis about 2.5 cm below the level of the laryngeal ventricle and laterally into the paralaryngeal fat with an erosion of the inner lamina of the thyroid cartilage. The mass grows around the cornu inferior of the thyroid cartilage with an extralaryngeal extension. Posteriorly, there was an invasion of the arytenoid and cricoid cartilage. Anteriorly, extension across the midline into the left true vocal cord was observed. Therefore, a T4 stage was diagnosed and a TLE was proposed and performed.

**Figure 1:**
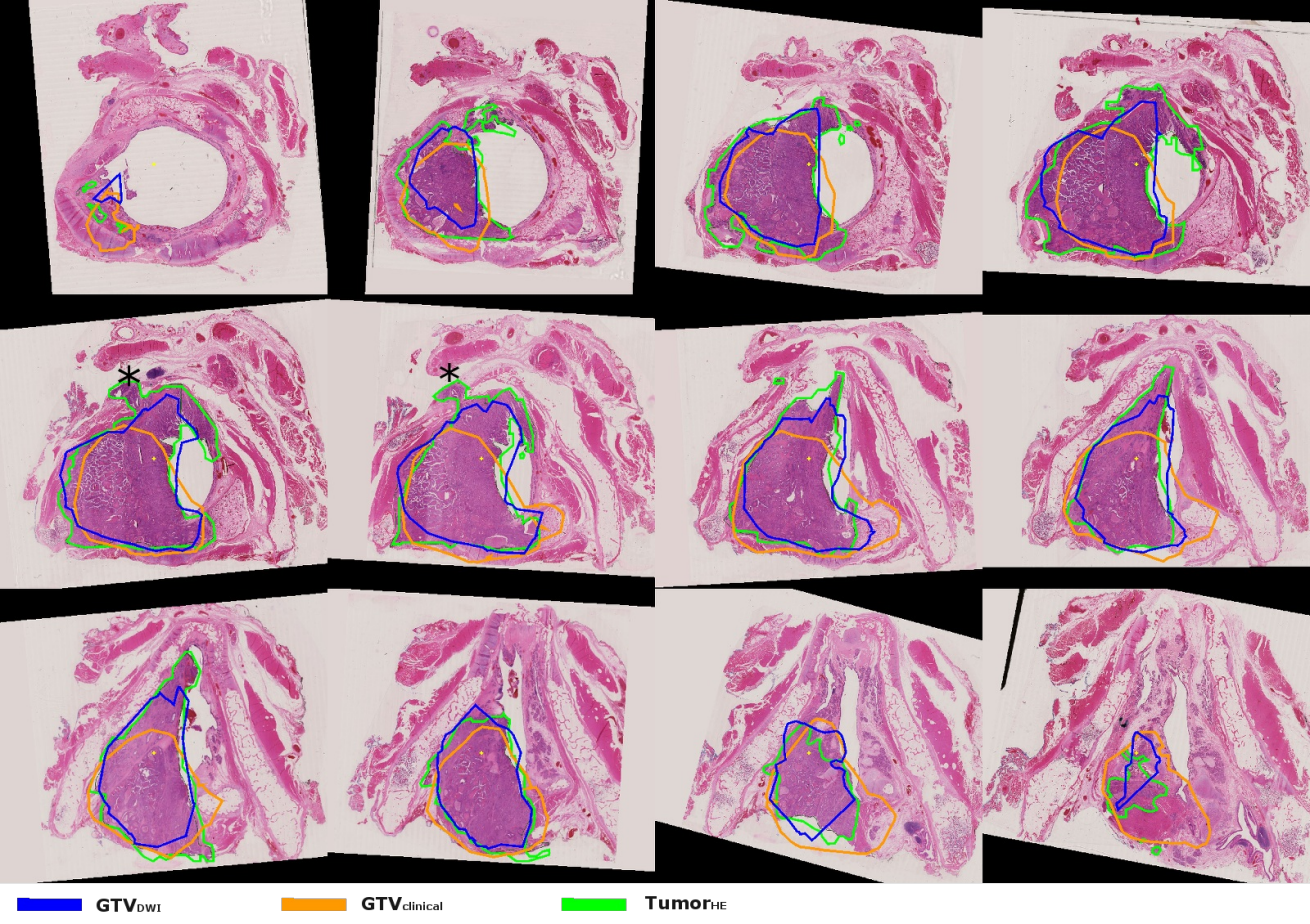
HE-stained sections with the larynx tumor of case I. The sections are shown from caudal to cranial direction (from left to right, top to bottom). Both GTV delineations are registered to the HE-stained sections. From these sections, it can be observed that at some locations, the tumor extent is overestimated and at other locations, it is underestimated. In this case, the underestimation is larger in the anterior direction, especially for the GTVclinica. The extralaryngeal growth between the thyroid and cricoid cartilage (indicated with *) and the anterior extent over the midline was not observed in both GTV delineations. The GTV delineations can extend into the air cavity due to small deformations of the specimen and registration errors. GTV: gross tumor volume; HE: hemotoxylin-eosin; tumor_HE_: microscopic tumor delineated by pathologist; GTV_clinical_: GTV delineated by radiation oncologist; GTV_DWI_: GTV semi-automatically delineated based on DWI

On histopathology, the tumor shows a predominantly cohesive growth pattern with a maximum tumor diameter of 4.8 cm. There is a bilateral extension of the tumor, but the main tumor bulk is located at the right side of the larynx. The HE sections show an extension through the cartilage and extralaryngeal extension (Figure 1).

**Figure 2:**
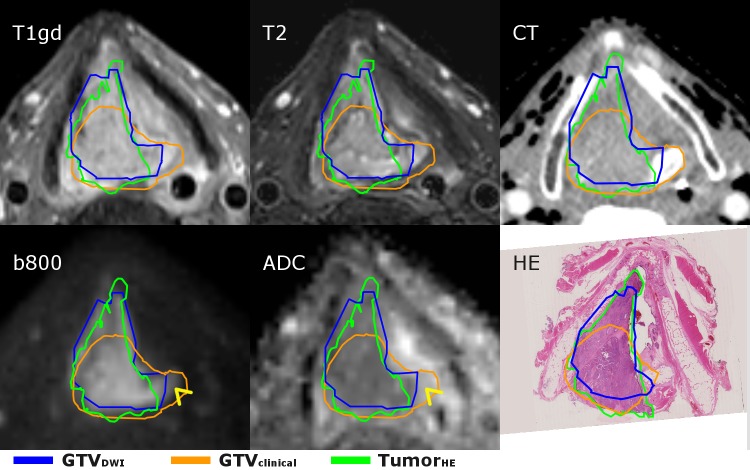
The transverse plane of the larynx tumor of case I for the various images. The tumor growth in the anterior direction is enhanced in the DWI image, while the extent is less visible on the other imaging. The GTVDWI was manually adapted when the DWI image and the ADC map both were enhanced (see arrowhead) because this is indicating edema instead of tumor tissue. The delineations differ slightly from the delineations shown in the HE section due to an angulation difference. GTV: gross tumor volume; HE: hemotoxylin-eosin; ADC: apparent diffusion coefficient; tumor_HE_: microscopic tumor delineated by pathologist; GTV_clinical_: GTV delineated by radiation oncologist; GTV_DWI_: GTV semi-automatically delineated based on DWI

The tumor_HE_ volume was 12.3 ml, and the GTV_clinical_ and GTV_DWI_ volumes were comparable with 12.5 ml and 11.7 ml, respectively. The conformity of tumor_HE_ with both GTVs was 57% and 70% for GTV_clinical_ and GTV_DWI_, respectively. The tumor_HE_ was included in the GTV_clinical_ and the GTV_DWI_ by 73% and 80%, whereas the tumor was overestimated at other regions because 72% and 84% of the GTVs overlapped with the tumor_HE_. The microscopic extent was underestimated with 7.5 mm (GTV_clinical_) and 3.0 mm (GTV_DWI_) (Figure 2).

Case II

The second case is a 68-year-old female diagnosed with a cT4aN2cM0 laryngeal carcinoma located around the epiglottis. The interval with surgery was six and eight days for CT and MRI, respectively.

On the b800 diffusion-weighted image, a clear and homogeneous contrast with high diffusion restriction between tumor and surrounding tissue was visible. The ADC map shows corresponding low ADC values in the tumor and increased ADC values directly adjacent to the tumor. This was in correspondence with the T1-weighted MRI, which showed a homogeneous contrast uptake within the tumor and an increased uptake adjacent to the tumor. The T2-weighted MRI cranially showed an increased homogeneous signal compared with the surrounding tissue, whereas more caudally, the contrast between the tumor and the surrounding tissue was low.

In the images, a large ulcerative mass on the right side of the larynx was visible, probably originating from the laryngeal side of the aryepiglottic fold. The maximum diameter measured in the coronal plane is 4.6 cm. There is an extension around the right side of the epiglottis. Caudally, extension into the false and true vocal cord is found, while a subglottic extension is not observed. There is an obliteration of the paralaryngeal fat with a broad contact with the thyroid cartilage. The thyroid cartilage shows some signal enhancement but no invasion was visible. Anteriorly, an extension across the midline at the level of the false vocal cord is evident. Cranially to the thyroid cartilage on the right, some extension beyond the larynx without invasion of the strap muscles was present. In addition, the tumor extended minimally through the laryngeal incisura. No tumor invasion of the arytenoid or cricoid cartilage was apparent. The clinical tumor staging was T4. The patient was offered a total laryngectomy.

On histopathology, a poorly differentiated supraglottic squamous cell carcinoma of the basaloid type was visible. The growth pattern was mainly cohesive, with a diameter of 3.6 cm and bilateral extension. The tumor extended between the hyoid and the ventral part of the thyroid cartilage. The hyoid cartilage was slightly invaded by the tumor, resulting in a pathological T4 stage.

**Figure 3:**
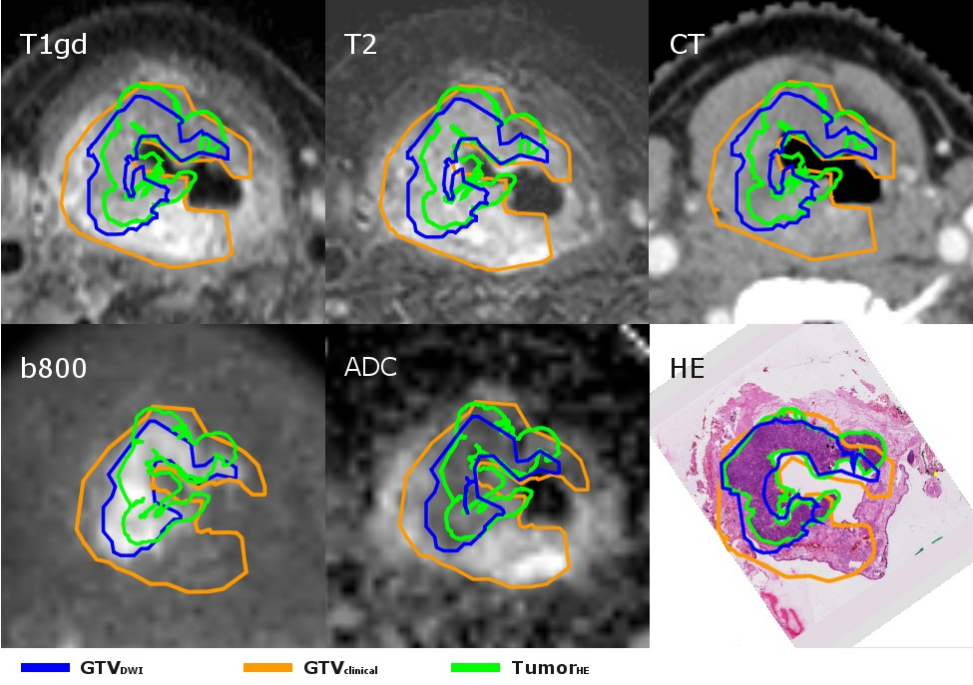
The transverse plane of the larynx tumor of case II for the various images. The GTVclinical overestimated the tumorHE, whereas the GTVDWI more closely resembles the tumorHE, especially in the posterior part of the larynx where tissue was excluded from the tumorHE and from the GTVDWI but included in the GTVclinical. This region was enhanced in the T2-weighted image, which indicates that these regions can be excluded from the GTVclinical based on these enhancements. GTV: gross tumor volume; HE: hemotoxylin-eosin; tumor_HE_: microscopic tumor delineated by pathologist; GTV_clinical_: GTV delineated by radiation oncologist; GTV_DWI_: GTV semi-automatically delineated based on DWI

The volume of this tumor measured on histopathology was 10.9 ml, which was largely overestimated by GTV_clinical_ with a volume of 21.6 ml, and comparable to the GTV_DWI_ of 11.5 ml (Figure 3). The clinical delineation showed more overestimation than the DWI segmentation (37% and 70% of the GTV_clinical_ and GTV_DWI_-contained tumor, respectively), while a comparable region of the tumor was included in both GTV delineations (72% and 74% for GTV_clinical_ and GTV_DWI_, respectively). The underestimation of the tumor extent by the GTV_clinical_ was 2.1 mm and for the GTV_DWI_, it was 2.7 mm (Figure 3).

Case III

The third case is a 68-year-old male with a cT4aN0M0 laryngeal tumor. The time interval between imaging and TLE is one and four days for CT and MRI, respectively. On the imaging, an enhancing mass with restricted diffusion was visible on the left true vocal cord.

In this patient, the tumor on DWI showed a poor heterogeneous patch-like contrast. Therefore, the automatic segmentation of the tumor was difficult and evaluated as not reliable. Also on T2-weighted MRI and T1-weighted MRI after gadolinium administration, the tumor was heterogeneous but more distinct from normal tissue. The tumor extended into the paralaryngeal fat with a broad interface with the thyroid cartilage. The adjacent thyroid cartilage showed edema and enhancement, which is suggestive of cartilage invasion. In addition, the cricoid cartilage was eroded on the left side. Caudally, there was an extension in the subglottis, 1.2 cm below the level of the laryngeal ventricle. An extralaryngeal extension was not observed and there were reactive edema and enhancement around the tumor.

**Figure 4:**
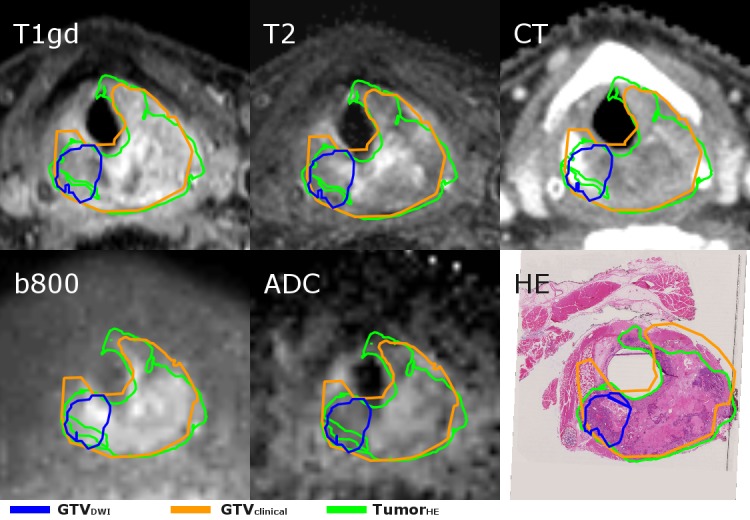
The transverse plane at the cricoid level of the larynx tumor of case III for the various images. The tumor is heterogeneous as can be seen from the HE section, with a mixture of cartilage, fibrotic tissue, and cohesively growing squamous tumor cells centrally located in the tumorHE. And, predominantly, cohesively growing squamous tumor cells at the sides, especially within the GTVDWI. This is reflected in the other images, where the enhancement within the tumor is inhomogeneous. The GTVDWI does not overlap with the whole tumorHE because of the higher ADC values in a part of the tumor. The GTVclinical shows a good overlap with the tumorHE. The delineations on the various images do vary in shape due to a difference in slice thickness, and the angulation of the HE sections compared to the imaging plane of the CT and MRI. GTV: gross tumor volume; HE: hemotoxylin-eosin; ADC: apparent diffusion coefficient; tumor_HE_: microscopic tumor delineated by pathologist; GTV_clinical_: GTV delineated by radiation oncologist; GTV_DWI_: GTV semi-automatically delineated based on DWI

On histopathology, a squamous cell carcinoma with an extension over the midline was visible, with a maximum diameter of 3.5 mm, predominantly cohesively growing. An extralaryngeal extension was observed at the dorsal and dorsolateral sides of the resection plane. Furthermore, focal perineural growth was observed. Erosion of the thyroid cartilage was not observed, while there was cartilage invasion in the cricoid (Figure 4). The tumor was histopathologically confirmed as stage T4.

The delineated volumes of tumor_HE_, GTVclinical, and GTV_DWI_, respectively, were 7.9 ml, 13.5 ml, and 7.1 ml. The conformity of both delineations with the tumor_HE_ was 48% and 19% for the GTV_clinical_ and the GTV_DWI_, respectively. The percentage of the tumor_HE_ included in the GTV_clinical_ and the GTV_DWI_ was 89% and 31%. The extent of the tumor was overestimated by both GTVs as 51% and 34% of the GTV_clinical_ and GTV_DWI_-contained tumor, respectively. In contrast, the microscopic tumor extent was underestimated in other regions, as the tumor_HE_ extended 1.5 mm from the GTV_clinical_ and 11.3 mm from the GTV_DWI_ (Figure 4).

## Discussion

In this case series study, GTV delineations based on clinical imaging (GTV_clinical_) and based on diffusion-weighted MRI (GTV_DWI_) were compared with the histopathological gold standard, the HE-delineated tumor. The validation of target definition on MRI is important because of the current developments in MR-guided radiotherapy with the integration of MRI with a radiotherapy treatment system.

In the various cases, different results in overlap and tumor extent outside the GTVs were observed. In case I and case II, the GTV_DWI_ resulted in a more concise target definition than the GTV_clinical_, with higher conformity. Although the delineated volume was smaller on GTV_DWI_, the extent of microscopic disease (tumor_HE_) outside the GTV was comparable to or smaller than the extent of microscopic disease outside the GTV_clinical_. In these two cases, it was demonstrated that a DWI-based target definition can give a fast and accurate tumor definition. However, in case III a large part of the caudal region of the tumor was missed by the GTV_DWI_ and included in the GTV_clinical_. The images of this case were difficult to interpret because of the heterogeneous signal intensity of the tumor in the DWI. Moreover, the contrast in the DWI was poor and the tumor segmentation was difficult to perform and was evaluated as unreliable. The poor results of this case show that when the tumor border is poorly visible on DWI, caution is urged, and a manual GTV delineation should be considered, including all MR images. Based on this single case, we suggest that a heterogeneous signal intensity on DWI with highly varying ADC values should be considered to include in the GTV, taking anatomical imaging into account. Whereas a more homogeneous ADC, as in case I, should be excluded from the GTV.

Furthermore, as a microscopic tumor was missed in GTV_DWI_, as was in the clinical GTV delineation, a clinical target volume (CTV) margin is required. GTV_DWI_ needed a small margin in case I and case II. A small margin in combination with a smaller initial GTV_DWI_ compared to GTV_clinical_ in cases I and II will result in a smaller CTV.

When a tumor with an acceptable contrast is found on DWI, GTV delineation based on DWI is less time-consuming than manual GTV delineation, which is important when online target definition is needed like on an integrated MRI-linac system.

In all three cases, the volume of the GTV_DWI_ was similar to the tumor_HE_ volume. In contrast, the tumor volume was overestimated in two cases by the GTV_clinical_. The large overestimation is in correspondence with the results for GTV delineation based on CT, MRI, or positron emission tomography (PET), as found in a previous study. Here, a large overestimation of the tumor volume was observed for all three modalities [6]. An overestimation of the tumor volume might lead to a higher dose in organs at risk (OARs) and an increased normal-tissue-complications probability (NTCP). Furthermore, a higher conformity with the tumor_HE_ and less overestimation might enable dose escalation in the GTV, which could result in a higher tumor control probability (TCP). In case II, an overestimation by the GTV_clinical_ could have been reduced when areas on T2-weighted and T1-weighted MR images with a higher intensity than the adjacent tumor bulk were excluded according to the delineation guidelines validated by Jager et al. [10].

The images of the presented cases were all acquired before the start of radiotherapy treatment. The contrast of the DWI scans might change during therapy because of several radiation effects on the tumor and surrounding tissue, such as edema, necrosis, and immune responses. These changes complicate the delineation of the tumor based on DWI scans. Besides, changes in ADC might have a predictive value for treatment response [7].

The presented three cases show the initial results of a larger ongoing DWI validation study. Patients in this study have a CT-PET scan and a complete MRI-exam with T1-weighted imaging and T2-weighted imaging. Based on this ongoing study, a general clinical target volume (CTV) margin can be derived for DWI to include the microscopic tumor extent. A further analysis of the performance in target definition and contrast will be performed.

## Conclusions

In this case series report, we demonstrated that DWI might be used for semi-automatic GTV delineation as a fast alternative for manual GTV delineation that was done by a comparison of both GTV delineations with the gold standard histopathology delineations. In two of the three cases, DWI-based GTV delineation resulted in an accurate delineation compared to the manual GTV delineation. In one case, the diffusion-weighted scan was of poor quality due to the biophysical properties of this tumor and was evaluated as unreliable for automatic segmentation. With careful use, DWI might be used for MR-guided radiotherapy treatment, like online target definition on an MRI-linac, when a clear contrast on DWI is visible. Further research with a larger sample size is needed to verify the results of this case series.
